# FTO Is a Relevant Factor for the Development of the Metabolic Syndrome in Mice

**DOI:** 10.1371/journal.pone.0105349

**Published:** 2014-08-21

**Authors:** Kathrin Ikels, Stefanie Kuschel, Julia Fischer, Wolfgang Kaisers, Daniel Eberhard, Ulrich Rüther

**Affiliations:** 1 Institute of Animal Developmental and Molecular Biology, Heinrich Heine University, Düsseldorf, Germany; 2 Institute of Metabolic Physiology, Heinrich Heine University, Düsseldorf, Germany; 3 Coordination Centre for Clinical Trials, Heinrich Heine University, Düsseldorf, Germany; Institut d’Investigacions Biomèdiques August Pi i Sunyer, Spain

## Abstract

The metabolic syndrome is a worldwide problem mainly caused by obesity. FTO was found to be a obesity-risk gene in humans and FTO deficiency in mice led to reduction in adipose tissue. Thus, FTO is an important factor for the development of obesity. Leptin-deficient mice are a well characterized model for analysing the metabolic syndrome. To determine the relevance of FTO for the development of the metabolic syndrome we analysed different parameters in combined homozygous deficient mice *(Lep^ob/ob^;Fto^−/−^). Lep^ob/ob^;Fto^−/−^* mice showed an improvement in analysed hallmarks of the metabolic syndrome in comparison to leptin-deficient mice wild type or heterozygous for Fto. *Lep^ob/ob^;Fto^−/−^* mice did not develop hyperglycaemia and showed an improved glucose tolerance. Furthermore, extension of beta-cell mass was prevented in *Lep^ob/ob^;Fto^−/−^*mice and accumulation of ectopic fat in the liver was reduced. In conclusion this study demonstrates that FTO deficiency has a protective effect not only on the development of obesity but also on the metabolic syndrome. Thus, FTO plays an important role in the development of metabolic disorders and is an interesting target for therapeutic agents.

## Introduction

The metabolic syndrome is a disease, which combines several different metabolic abnormalities such as central obesity, hypertension, elevated serum triglycerides and insulin resistance. Furthermore, it is strongly associated with ectopic fat accumulation, dysfunctions of muscle, adipose tissue and liver as well as pancreatic abnormalities. Therefore it is one of the major risk factors for cardiovascular diseases and diabetes [1], [2], [3], [4], [5]. Obesity is the most dominant factor for the development of the metabolic syndrome leading to insulin resistance and type 2 diabetes. Particularly obesity in childhood is strongly associated with the development of the metabolic syndrome [6], [7], [8]. Besides lifestyle and environmental factors, such as eating behaviour and physical activity, genetic predispositions play a basic role in the development of obesity.

In general, control of energy homeostasis is based on the regulation of food intake. This regulation mainly takes place in the hypothalamus. Especially two populations of neurons expressing NPY/AgRP and POMC/CART in the arcuate nucleus play an important role in the regulation of food intake. One of the appetite regulating proteins is leptin, a circulating hormone, released by adipocytes [9], [10]. It acts via binding to leptin receptors in the hypothalamus and thus inhibiting appetite stimulating neuropeptides NPY and AgRP on the one hand and activating appetite suppressing transmitters POMC and CART on the other hand [11], [12], [13]. Consequently, leptin deficiency leads to hyperphagia, resulting in severe obesity and hyperinsulinemia in human [14],[15],[16],[17].

The murine *Lep^ob/ob^* model is well characterized. Mice lacking leptin suffer from enormous weight gain, hyperglycaemia, hyperinsulinemia and glucose intolerance [18]. Weight loss can be induced by leptin infusion in *Lep^ob/ob^* mice. This leads to a reduction of appetite and therefore to a reduction of food intake [19], [20], [21]. *Lep^ob/ob^* mice have been widely used to study the details of the metabolic syndrome [18], [22], [23]. Furthermore, influences on the development of the metabolic syndrome by different means such as genetic factors or drugs have been addressed in the *Lep^ob/ob^* model [24], [25], [26], [27].

Genome-wide association studies (GWAS) of obese people indicated about 50 genes to be involved in body weight regulation [28]. One of these genes is the *Fat mass and obesity associated* gene *FTO*. In several studies, SNPs in the *FTO* gene were highly associated with increased body mass index. These studies could show that individuals carrying a risk allele exhibit about 1.5 kg higher weight than those with non-risk alleles most likely due to an increase in food intake [29], [30], [31], [32]. Consequently, the relation of leptin to FTO was addressed in further studies. Whereas one study could show that leptin downregulates FTO [33] another one provided evidence for a regulation of leptin signaling by FTO [34]. Thus, loss of FTO should result in a loss of leptin signaling and therefore hyperphagia.

Further characterization of FTO function by generating *Fto^−/−^* mice revealed FTO as an elementary protein in regulating energy homeostasis. In previous studies, we and others showed that loss of *Fto* leads to postnatal growth retardation and reduction in weight [35], [36]. Despite relative hyperphagia the leanness of *Fto^−/−^* mice seems to be a consequence of increased energy expenditure [36] which is accompanied by browning of epigonadal and inguinal white adipose tissue [37]. Another study confirmed reduced fat mass, increased food intake and energy expenditure [38]. However, adult onset FTO-deficient mice exhibited reduced body weight and lean mass but did not show changes in fat mass, food intake and energy expenditure [35]. As FTO has a function in energy homeostasis we were interested to see whether FTO is also important for the development of the metabolic syndrome. To address this, we used the well characterised leptin-deficient mouse model (*Lep^ob/ob^*). *Lep^ob/ob^;Fto^−/−^* mice were analysed and compared to *Lep^+/+^; Fto^+/+^*, *Lep^ob/ob^;Fto^+/+^* and *Lep^ob/ob^;Fto^+/−^* littermates.

## Methods

### Ethics statement

All animal experiments were performed in accordance with the relevant national guidelines for the Care and Use of Laboratory Animals (LANUV) and with approval from the authority for animal work at Heinrich Heine University Düsseldorf, Germany (Permit number 84-02.05.20.11.217).

### Animal care

C57BL/6J *Lep^ob/ob^* mice were kindly provided by Jens Brüning, Cologne. *Fto* mutant mice (C57BL/6J) are described [36]. *Fto* ^+/−^ mice were crossed with *Lep^ob/+^* mice and afterwards *Lep^ob/+^*;*Fto* ^+/−^ mice were crossed among each other. All mice used in this study were housed at 22–24°C on a 12/12 h dark-light cycle with food and water ad libitum. Body weight was measured every week over a period from 3 to 30 weeks. At the age of 30 weeks animals were killed, body length was measured, organs and fat depots were isolated and weighed. Organs needed for further experiments were stored or handled according to the method.

### Antibodies

We used a polyclonal antibody against insulin raised in rabbit (Santa Cruz Biotechnology, sc-9168) and a polyclonal antibody against glucagon raised in guinea pig (Millipore, 4031-01F). As secondary antibodies Cy2-conjugated IgGs raised in guinea pig (Jackson Immuno Research, # 706-225-148) and Cy3-conjugated IgGs raised in rabbit (Jackson Immuno Research, #711-165-152) were used.

### Blood analysis

Blood glucose was determined from whole blood using an automatic monitoring system (GlucoMen Visio Sensor, A. Menarini Diagnostics). Insulin was measured in blood plasma using Ultra Sensitive Rat Insulin ELISA Kit (Chrystal Chem. Inc, #90060).

### Body composition analysis

Lean mass and fat mass of living animals was determined using an NMR Analyser (Minispec, Bruker).

### Cell size measuerements of adipocytes

Paraffin embedded epigonadal adipose tissue sections (12 µm) were stained with Hematoxylin and Eosin and photographed (Axiocam MRc, Zeiss). Area of 300 adipocytes per individual were measured using Axiovision Rel. 4.8 (Zeiss).

### Extraction of fat from the liver

Nine pieces of liver (every piece about 50 mg) were isolated, weighed and homogenized in water and transferred into glass tubes. 4 ml chloroform/methanol (2∶1) was added, mixed and incubated over night at 4°C to separate the phases. The next day 0.8 ml 0.9% NaCl was added and mixed again, followed by a centrifugation step (10 minutes at 2500 rpm). The lowermost phase of 3 samples of the same individual was transferred into a new glass vial, which has been weighed before. Over three days the liquid evaporated. The tube with the remaining fat was weighed again and the amount of fat per mg liver was determined. A triple quantification has been performed in every case.

### Glucose and Insulin tolerance test

Glucose tolerance tests were performed on animals fasted for 16 h. Insulin tests were performed on randomly fed animals. Either 2 g kg^−1^ (at the age of 6 weeks) body weight of a 20% glucose solution (1 g kg^−1^ at the age of 14 weeks) or 0.75 U kg^−1^ body weight of human regular insulin (Actrapid, Novo Nordisk, diluted to 100 mU/ml) was injected into the peritoneal cavity and blood glucose levels were determined at defined time points (after 15, 30, 60, 120 minutes for GTT, after 15, 30, 60 minutes for ITT).

### Immunofluorescence

Pancreas cryo sections were washed with PBS, permeabilized with PBS/0.1% Triton-X-100 and blocked with 10% FCS in PBS/0.1% Triton-X-100. Afterwards, they were incubated with primary antibodies (diluted in blocking solution) over night at 4°C. Beta cells were illustrated by insulin staining, alphacells were illustrated by glucagon staining. After 3 washing steps they were incubated with secondary antibodies (diluted in blocking solution) for 4–6 hours, washed again and embedded in Mowiol containing DAPI (Merck, #1.24653).

### Islets measurements

To measure the area of islets of Langerhans, pancreas sections were stained for insulin and photographed (Axiocam MRM, Zeiss). Then the stained islets were surrounded and the areas were determined with a documentation software (Axiovision Rel. 4.8, Zeiss). Additionally, the area of the whole pancreas section was photographed (Axiocam MRC, Zeiss), measured and the islet area in relation to the whole area was determined (Axiovision Rel. 4.7, Zeiss). For each individual 9 sections were measured which was about every 20th section.

### Metabolic data

Physical activity and food intake was measured using the metabolic cages (PhenoMaster, TSE-System). Animals were kept for 72 hours separated for adaptation in metabolic cages without measurement. After adaption activity and food intake was monitored for 72 hours. Activity was recorded using infrared sensor frames. Interruptions of infrared sensors were detected by a control unit and registered by a computer with the relevant software (ActiMot2, TSE Systems). Food intake was measured by integrated weighting sensors.

### Oil red O staining

For staining the stock solution (Oil red O (Sigma, #O-9755) dissolved in propanol) was diluted in water (working solution) and filtered. Liver cryo sections were incubated with the working solution for 10 minutes, washed under running water and afterwards incubated with hematoxylin for one minute. After a second washing step the sections were embedded in glycerol gelatine and sealed.

### Real-time PCR Analysis

RNA from epigonadal adipose tissue was isolated using RNeasy Kit (Qiagen #74104) and RNase-Free DNase Set (Qiagen # 79254). Isolated RNA was converted into cDNA by using Expand Reverse Transcriptase (Roche # 11785826001). Quantitative Real-time PCR was performed by employing a Mx3000P qPCR System (Agilent Technologies) and by using the Agilent Brilliant III Sybr green kit. The following Primers (MWG Eurofins) were used: *RPLP0* (5′: GATGCCCAGGGAAGACA, 3′: ACAATGAAGCATTTTGGA), *PPARγ2* (5′:GTTTTATGCTGTTATGGGTG, 3′:GTAATTTCTTGTGAAGTGCTCATAG), *Adiponectin* (5′:TGTACGATTGTCAGTGGATCTG, 3′:ACGTCATCTTCGGCATGACT), *TNFα* (5′:TCTTCTCATTCCTGCTTGTGG, 3′:GGTCTGGGCCATAGAACTGA), *MCP1* (5′:CATCCACGTGTTGGCTCA, 3′:GATCATCTTGCTGGTGAATGAGT), *IL-6* (5′:AACGATGATGCACTTGCAGA, 3′:GAGCATTGGAAATTGGGGTA), *UCP1* (5′: ACTGCCACACCTCCAGTCATT, 3′:CTTTGCCTCACTCAGGATTGG). Gene activity was expressed in relation to the housekeeping gene *RPLR0* using the ddct method.

### Tissue embedding

For cryo sections organs were fixed in 4% paraformaldehyde (PFA) and incubated in 30% sucrose (in PBS) over night at 4°C. After that they were embedded in Tissue-Tek O.C.T. (Sacura Finetec, # 4583) and stored at −80°C. For analysis 7–12 µm thin cryostat sections were prepared.

For paraffin sections organs were fixed in 4% PFA over night. Then they were dehydrated by increasing concentrations of ethanol and butanol, embedded in paraffin and sectioned (7–12 µm). For histological analyses the sections were stained with hematoxylin and eosin and embedded in entellan (Merck, # 1.07961).

### Statistical Data

All values are presented as mean ± SEM. Statistical analysis was performed using R (version 3.1.0) [39]. Differences between two samples were tested by two-sided t-Test. Differences between all present genotypes were determined by ANOVA (aov) followed by construction of confidence intervals using Tukey's Honest Significant Difference method (TukeyHSD). Time coures of blood glucose levels were compared by bootstrapped mean values for each point of time (10000 samplings). Timely dependent weight gains were modeled using local polynomial regression (loess). Confidence intervals were calculated by bootstrapping loess regression (10000 samplings).

## Results

### Influence of FTO on body weight development in *Lep^ob/ob^* mice

**Figure 1:**
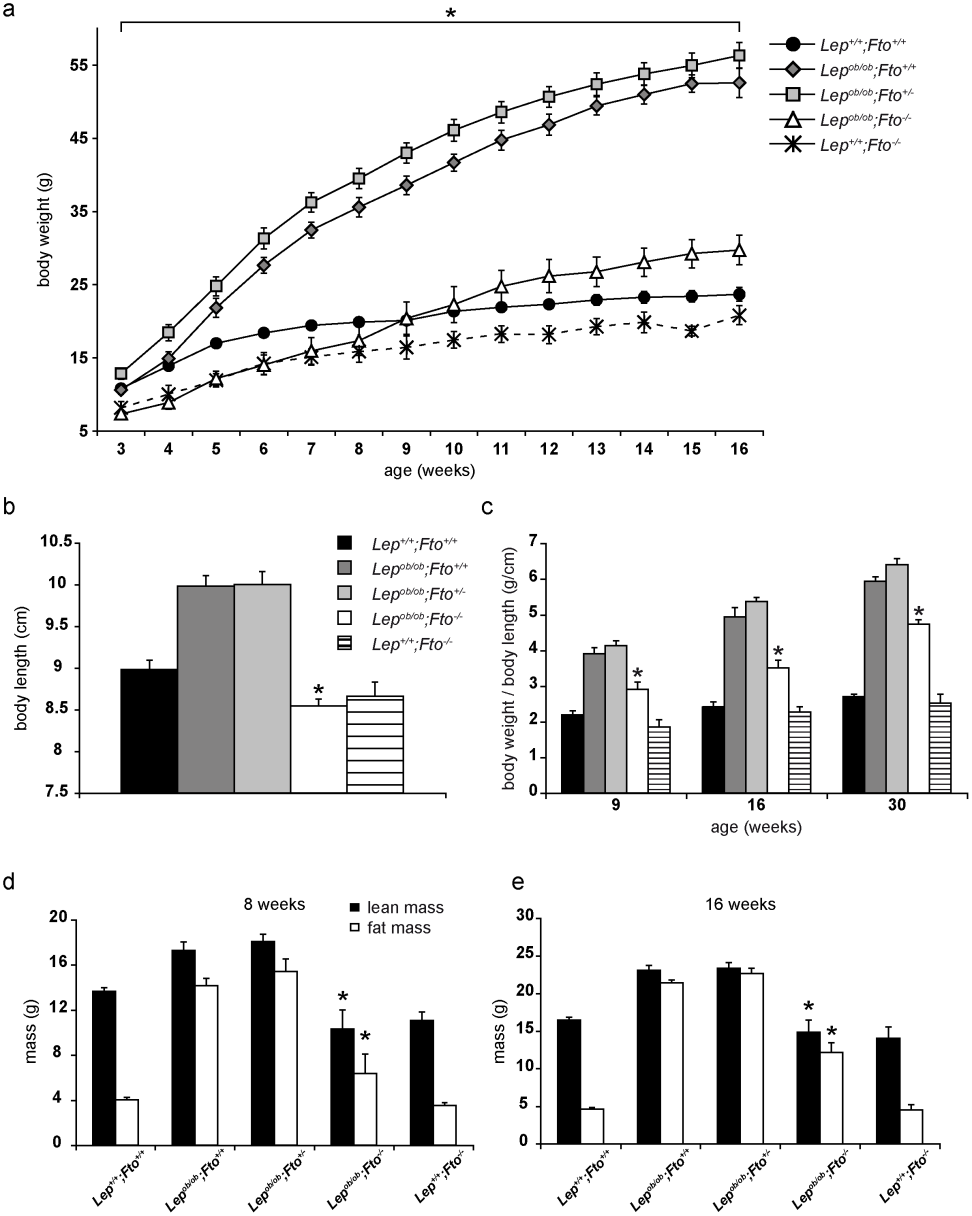
FTO contributes to gain of body weight in *Lep^ob/ob^* mice. All data are collected from female mice. a) Development of body weight from 3 to 16 weeks of age. n(*Lep^+/+^; Fto^+/+^*) = 15–20, n(*Lep^ob/ob^;Fto^+/+^*) = 11–13, n(*Lep^ob/ob^;Fto^+/−^*) = 15–19, n(*Lep^ob/ob^;Fto^−/−^*) = 9–12, n(*Lep^+/+^;Fto^−/−^*) = 4–6. Asterisks (*) indicate significant p-values between *Lep^ob/ob^;Fto^−/−^* and *Lep^ob/ob^;Fto^+/+^*. Further significant p-values were found: *Lep^+/+^;Fto^+/+^*–*Lep^ob/ob^;Fto^−/−^*: p≤0,05 (weeks 3–8+11–16); *Lep^+/+^;Fto^+/+^*–*Lep^ob/ob^;Fto^+/+^*: p≤0,05 (weeks 4–16); *Lep^+/+^;Fto^+/+^*–*Lep^ob/ob^;Fto^+/−^*: p≤0,05 (weeks 4–16); *Lep^ob/ob^;Fto^−/−^*–*Lep^ob/ob^;Fto^+/−^*: p≤0,05 (weeks 3–16); *Lep^ob/ob^;Fto^+/+^*–*Lep^ob/ob^;Fto^+/−^*: p≤0,05 (weeks 4–14);; *Lep^+/+^;Fto^+/+^*–*Lep^+/+^;Fto^−/−^*: p≤0,05 (weeks 3–16). b–e) *indicate significant p-values to *Lep^ob/ob^;Fto^+/+^*. b) Body length at the age of 30 weeks (n = 14, 15, 17, 15, 10). c) Body weight in relation to body length at the age of 9 (n = 6, 6, 7, 5, 5), 16 weeks (n = 4, 6, 7, 5, 4) and 30 weeks (n = 14, 15, 17, 15, 10) of age. d) Lean mass and fat mass at the age of 8 weeks (n = 7, 8, 4, 4, 3). e) Lean mass and fat mass at the age of 16 weeks (n = 12, 7, 9, 4, 3). All data are presented as mean. Error bars indicate the SEM.

The extreme body weight is the most obvious phenotype of leptin deficiency. Therefore, we firstly analysed the influence of FTO on body weight gain in *Lep^ob/ob^* mice within the first 16 weeks of their life. Since more female than male mice were available for the analysis due to a 50% higher postnatal death rate of males (Figure S1) the data are presented for females. Nevertheless, as presented in Figure S2 the data are very similar for male mice. *Lep^ob/ob^;Fto^+/+^* mice as well as *Lep^ob/ob^;Fto^+/−^* mice gained more weight in a shorter time than wild type mice did, while absence of FTO and leptin resulted in an increase in body weight that was similar to wildtype after 9 weeks (Fig. 1a). In the first 8 weeks of their life, however, these *Lep^ob/ob^; Fto^−/−^* mice were similar to the *Fto^−/−^* mice (Fig. 1a). Thus, FTO contributes to body weight development in *Lep^ob/ob^* mice. Since it was described that *Fto^−/−^* mice also have a reduced body length we addressed this phenomenon in *Lep^ob/ob^* as well. As published *Lep^ob/ob^* individuals displayed an increased body length compared to wild type mice (Fig. 1b). Interestingly, the loss of FTO was dominant over the loss of leptin resulting in leptin-deficient mice being similar to the *Fto^−/−^* mice (Fig. 1b). Considering this, we adjusted the body weight to body length for 9 weeks and 16 weeks old mice. Nevertheless, mice deficient for leptin and FTO were either equal to wild type (9 weeks old) or, when 16 weeks old, 25% heavier than wild type (Fig. 1c). Since this finding suggested an age dependent body weight change in *Lep^ob/ob^;Fto^−/−^* mice we investigated 30 weeks old mice. As shown in Fig. 1c, even in these mice a significant difference between *Lep^ob/ob^;Fto^+/+^* mice and *Lep^ob/ob^;Fto^−/−^* mice was obvious.

Next we analysed body composition of all five genotypes. In 8 weeks old *Lep^ob/ob^;Fto^+/+^* mice lean and fat mass were significantly increased compared to wild type mice while *Lep^ob/ob^;Fto^−/−^* mice showed no significant differences (Fig. 1d). In 16 weeks old mice all *Lep^ob/ob^* mice independent of the *Fto* genotype showed a significant increase in fat mass compared to wild type mice. However, only the *Lep^ob/ob^* mice with FTO additionally displayed an increase of lean mass (Fig. 1e). Thus, early in life FTO contributes to lean and fat mass development in *Lep^ob/ob^* mice.

**Figure 2:**
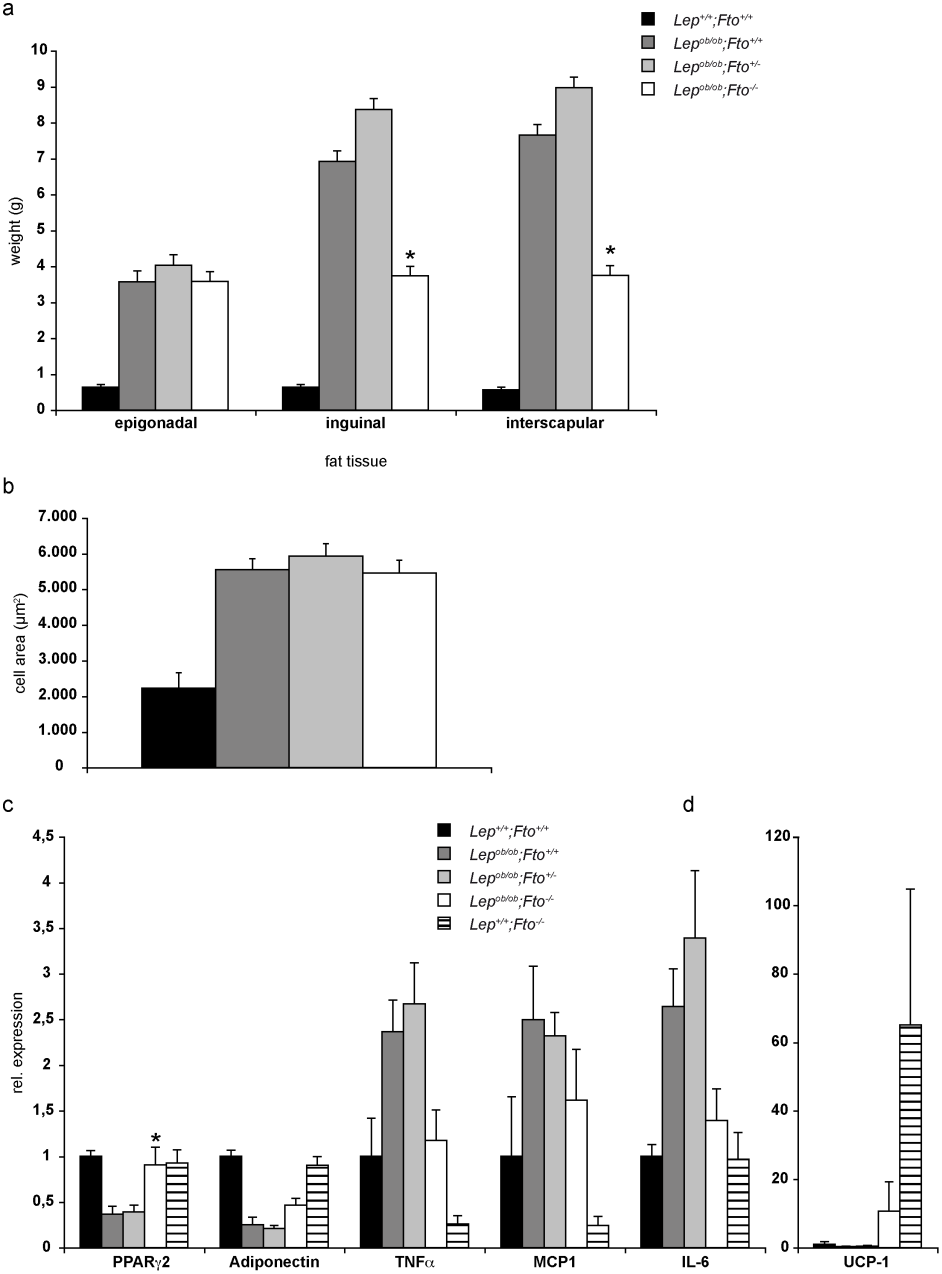
Detailed analysis of adipose tissue. All data are collected from 30 weeks old mice. *indicate significant p-values between *Lep^ob/ob^;Fto^+/+^* and *Lep^ob/ob^; Fto^−/−^*. a) Weights of different fat pads from female mice (n = 13, 16, 18, 16). b) Area size of epigonadal fat cells from female mice (n = 4, 4, 8, 7). c+d) Expression analysis for different marker genes of epigonadal adipose tissue (n = 6, 4, 5, 5, 5). Following p-values were calculated: between *Lep^ob/ob^;Fto^+/−^* and *Lep^ob/ob^; Fto^−/−^*: PPARγ2: p = 0,08, Adiponectin: p = 0,21, TNFα:p = 0,06, IL-6:p = 0,03. Data are presented as mean. Error bars indicate the SEM.

In contrast to inguinal and interscapular fat, the epigonadal fat tissue of 30 weeks old female mice did not differ much in weight between the three *Lep^ob/ob^* genotypes (Fig. 2a). Therefore, we analysed this tissue in more details. Firstly, adipocyte cell size of leptin deficient mice did not differ FTO dependently (Fig. 2b). However, analysis of adipose marker gene expression in *Lep^ob/ob^;Fto^−/−^* mice revealed interesting differences (Fig. 2c). Genes encoding for proteins which are involved in inflammation (TNF*α*, MCP1, IL6) were similar to wild type mice while expression of PPARγ2 and adiponectin was equal or lower compared to wild type but higher compared to the other leptin deficient mice. Browning of white fat was indicated by increased expression of UCP-1, however not as strong as in Fto-negative mice (Fig. 2d). Thus, loss of Fto in *Lep^ob/ob^* mice leads to a normalisation of the adipogenic program and to a reduction of inflammation. Furthermore, a transformation of white to brown adipose tissue seems to happen.

Next we analysed food intake. As expected *Lep^ob/ob^* mice with FTO fed twice as much as wild type mice (Figure S3a). *Lep^ob/ob^* mice without FTO however, fed less than wild type mice although the difference was not significant even when the lean mass of the animals were considered (Figure S3a,b). Obesity is the consequence of an imbalance of food intake and physical activity. Our data clearly showed that all *Lep^ob/ob^* mice independent of the Fto genotype showed equivalent low physical activity levels (Figure S3c). Thus, hyperphagia of *Lep^ob/ob^* mice is positively influenced by FTO.

### FTO contributes to the development of hyperglycaemia

**Figure 3:**
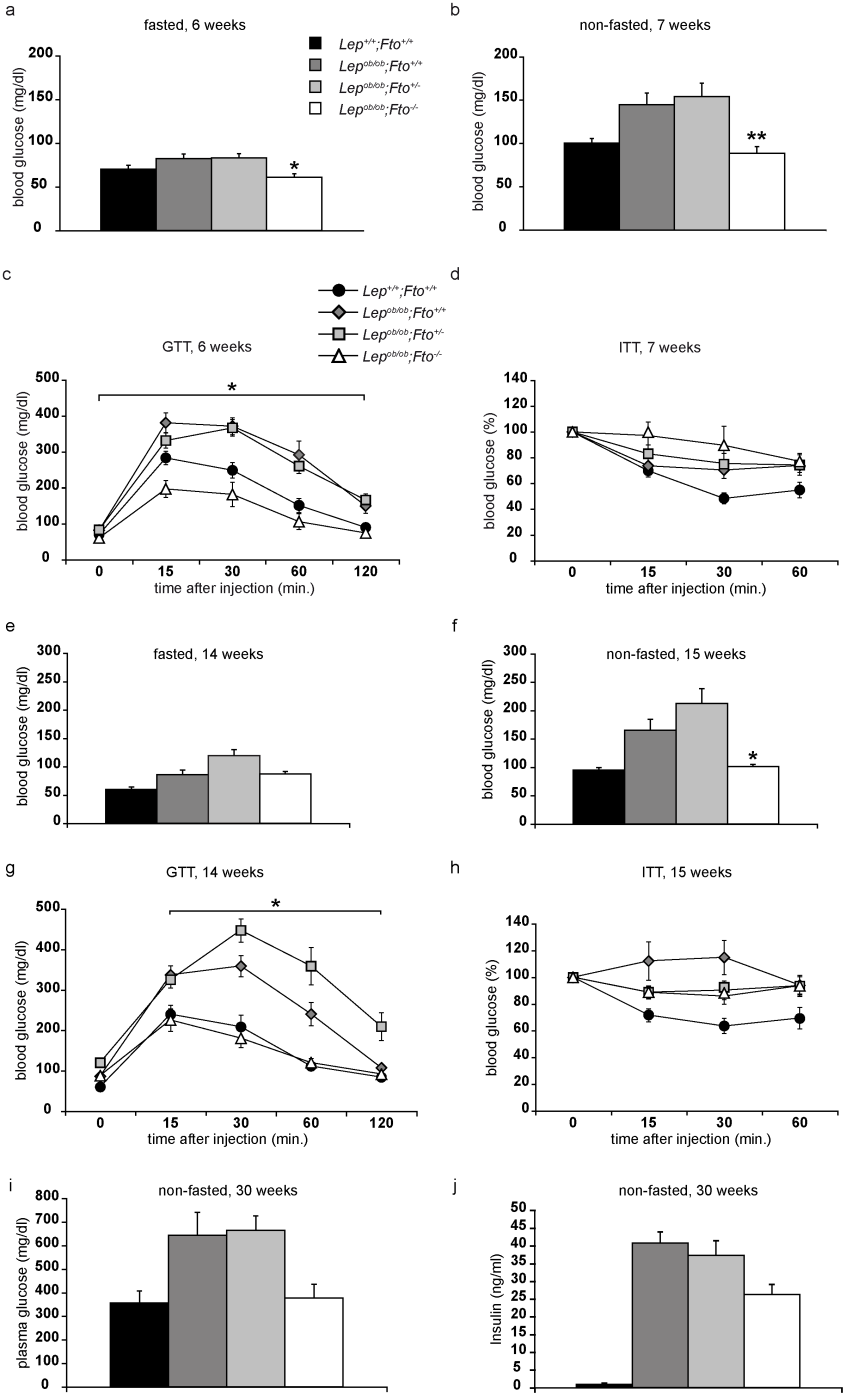
FTO contributes to the development of hyperglycaemia. All data are elevated from female mice. *indicate significant p-values between *Lep^ob/ob^;Fto^+/+^*and *Lep^ob/ob^;Fto^−/−^*. a) Fasted glucose levels in mice at the age of 6 weeks (n = 13, 10, 12, 8) b) Non-fasted glucose level at the age of 7 weeks (n = 12, 9, 12, 9). c) Glucose tolerance test (GTT) at the age of 6 weeks (n = 13, 10, 12, 8). d) Insulin tolerance test (ITT) at the age of 7 weeks (n = 12, 9, 12, 9). Glucose values are presented relative to initial glucose levels. e) Fasted glucose levels in mice at the age of 14 weeks (n = 11, 7, 9, 9). f) Non-fasted glucose level at the age of 15 weeks (n = 11, 8, 14, 12). g) GTT at the age of 14 weeks (n = 11, 7, 9, 9). h) ITT at the age of 15 weeks (n = 11, 8, 14, 12). Glucose values are presented relative to initial glucose levels. i) Plasma glucose levels of 30 weeks old non fasted mice (n = 8, 5, 5, 6). j) Plasma insulin levels of non-fasted mice at the age of 30 weeks (n = 3, 3, 7, 3). All data are presented as mean. Error bars indicate the SEM.

As increased body weight can result in elevated glucose levels, we performed glucose tolerance tests (GTT). We chose mice at the age of 6 weeks because body weight differences were already apparent (Fig. 1a). In fasting mice, glucose levels were already higher in *Lep^ob/ob^;Fto^+/+^* and *Lep^ob/ob^;Fto^+/−^* mice compared to wild type and *Lep^ob/ob^;Fto^−/−^* mice (Fig. 3a). Consistently, in GTT, *Lep^ob/ob^;Fto^−/−^* mice had already normalised their glucose levels after 60 minutes, the *Lep^ob/ob^* mice, being either wild type or heterozygous for *Fto*, continued to have elevated levels even after 120 minutes (Fig. 3c). These levels are very similar to the levels of blood glucose of non-fasted mice at the same age (Fig. 3b). Thus, leptin-deficient mice with FTO display a hyperglycaemia already early in life. To analyse the responsiveness to insulin, we chose the same mice a week later for the insulin tolerance test (ITT). Whereas wild type mice reduced their glucose levels within 30 minutes to about 50%, glucose levels of all *Lep^ob/ob^* mice were only reduced to about 80% after 30 minutes (Fig. 3d). However, *Lep^ob/ob^;Fto^−/−^* mice showed lower starting glucose concentrations (Fig. 3b). The further development of the hyperglycaemia was addressed by repeating both assays 8 weeks later. At the age of 14 weeks the GTT was comparable to the one performed in 6 weeks old mice (compare Fig. 3c and Fig. 3g). Thus, development of hyperglycaemia in *Lep^ob/ob^* mice is depending on FTO. However, in the ITT performed with mice at the age of 15 weeks, differences become obvious. Only wild type mice reduced glucose levels upon insulin injection (Fig. 3h). In contrast, all *Lep^ob/ob^* mice did not respond to the insulin injection (Fig. 3h) suggesting an insulin resistance at that age. Nevertheless, *Lep^ob/ob^;Fto^−/−^* mice still displayed lower starting glucose concentrations (Fig. 3 f). When we killed the mice at the age of 30 weeks we analysed glucose (Fig. 3i) and insulin (Fig. 3j) concentration in plasma. All leptin-deficient mice had at least 10 fold higher levels compared to wild type, however plasma glucose is not increased in *Lep^ob/ob^;Fto^−/−^* mice. Thus *Lep^ob/ob^;Fto^−/−^* mice can handle high glucose levels, although they are insulin resistant.

### Increase of pancreatic islet size in leptin-deficient mice is dependent on FTO

**Figure 4:**
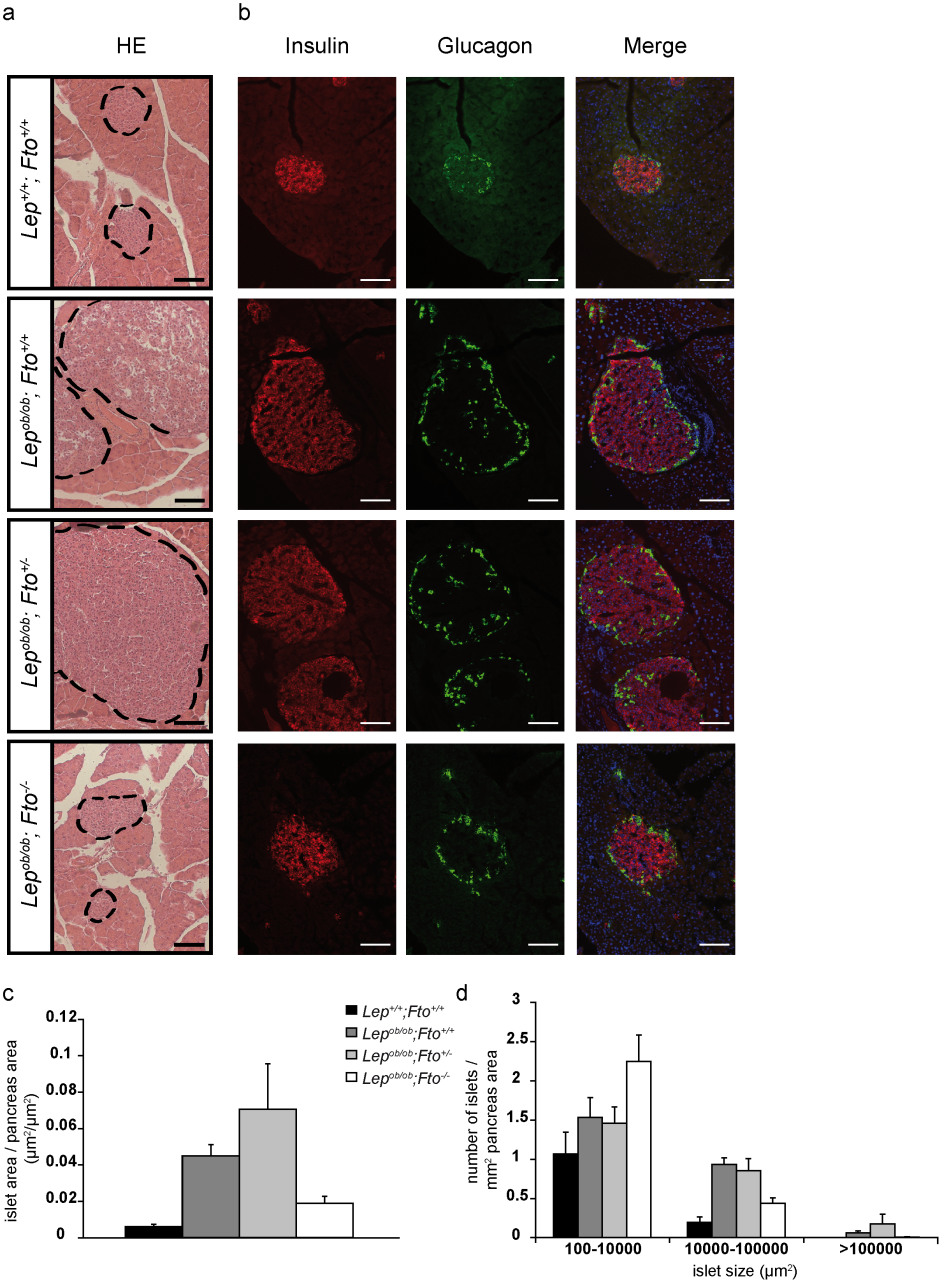
Increase of pancreatic islet size in leptin-deficient mice is depending on FTO. a) Haematoxylin and eosin staining on paraffin sections of the pancreas for all 4 genotypes. b) Immunofluorescence staining for insulin (red) and glucagon (green) on pancreas sections. Nuclei are stained with DAPI. Scale = 100 µm. c) Area of beta cells in relation to full pancreas section area. d) Number of islets of Langerhans which were found on sections categorized into 3 groups of size. Pancreas were taken from 30 weeks old female mice (n = 5, 6, 7, 5). All data are presented as mean. Error bars indicate the SEM.

The increased demand for the production of insulin during insulin resistance and hyperglycemia can result to beta cell hypertrophy and proliferation, and finally to enlarged islets. To analyse islet growth, we chose 30 weeks old animals, isolated the pancreas and stained either for histology or for alpha or beta cells in the islets with antibodies directed against glucagon or insulin. Size measurements of islets of Langerhans showed an increase in size and the appearance of enormous islets in the pancreas of *Lep^ob/ob^;Fto^+/+^* and *Lep^ob/ob^;Fto^+/−^* animals (Fig. 4a+c). They possessed more islets of a size of 10000–100000 µm^2^ than wild types and even islets of more than 100000 µm^2^ can develop which were never seen in wild type mice. This phenotype of increased islet sizes was found to be reduced in *Lep^ob/ob^;Fto^−/−^* mice (Fig. 4a–d). Although *Lep^ob/ob^;Fto^−/−^* mice had bigger islet areas per pancreas area than wild type, they had less islet areas than *Lep^ob/ob^;Fto^+/+^* and *Lep^ob/ob^;Fto^+/−^* animals (Fig. 4c). Furthermore, in *Lep^ob/ob^;Fto^−/−^* mice size distribution was altered and they never showed giant islets (Fig. 4d).

### Increased lipid content in liver cells of leptin-deficient mice is influenced by FTO

Excess of lipids in the blood leads to dysregulation of energy storage and ectopic fat accumulation within and around different organs. This increased accumulation can impair the function of these organs and thus interfere with the health status of individuals. The liver is one of these organs, in particular in older mice. Increased fat accumulation in the liver can lead to development of hepatic insulin resistance. Therefore, the same mice as used for the pancreas analyses were chosen.

**Figure 5:**
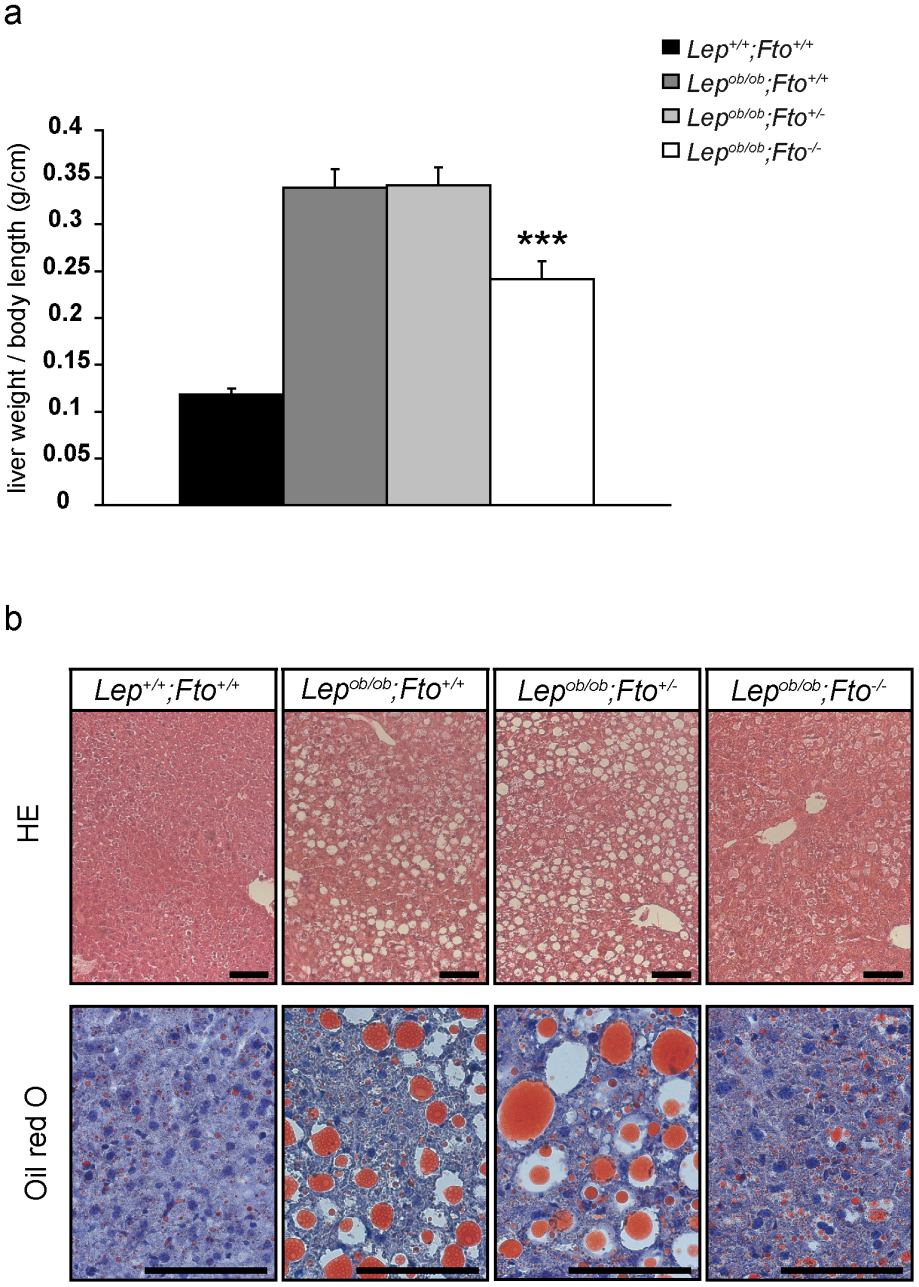
Accumulation of fat in liver of leptin-deficient mice is influenced by FTO. a) Liver weight in relation to body length (n = 13, 14, 16, 14). *indicate significant p-values between *Lep^ob/ob^;Fto^+/+^*and *Lep^ob/ob^;Fto^−/−^*. b) Haematoxylin and eosin staining on paraffin sections (upper row) and oil red O staining on cryo sections (lower row) for all 4 genotypes. Scale = 100 µm. Livers were used from female mice at the age of 30 weeks. All data are presented as mean. Error bars indicate the SEM.

Liver weight in leptin-deficient mice showed a dramatic increase in comparison to organs of wild type mice (Fig. 5a). The loss of FTO in leptin-deficient animals led to a clear reduction in liver weight in comparison to *Lep^ob/ob^;Fto^+/+^* and *Lep^ob/ob^;Fto^+/−^* littermates, but they still showed a higher weight than wild type animals (Figure S4a). *Lep^ob/ob^;Fto^−/−^* mice were significantly smaller than their littermates, therefore liver weights were calculated per body length. Nevertheless, livers were lighter in *Lep^ob/ob^;Fto^−/−^* mice compared to *Lep^ob/ob^;Fto^+/+^* and *Lep^ob/ob^;Fto^+/−^* littermates (Fig. 5a).

To investigate the degree of steatosis in the liver, we isolated triglycerides of the organs and measured the amount by weight. Obviously loss of FTO led to a significant decreased fat accumulation in the liver of leptin-deficient mice (Figure S4b). This was also visible in histological analyses on paraffin liver sections showing a more homogeneous area in *Lep^ob/ob^;Fto^−/−^* mice compared to *Lep^ob/ob^;Fto^+/+^* and *Lep^ob/ob^;Fto^+/−^* littermates (Fig. 5b). Furthermore, an Oil red O staining indicated a reduction of fat droplets in *Lep^ob/ob^;Fto^−/−^* mice (Fig. 5b). Thus, accumulation of fat in the liver of leptin-deficient mice depends on FTO.

## Discussion

In this study, we could show that FTO is a factor which contributes substantially to the development of the obese phenotype in leptin-deficient mice and thus to the metabolic syndrome as exemplified by hyperglycaemia and increased lipid content in liver.

### FTO and development of obesity

Our analyses of body weight development of leptin-deficient mice within the first 10 weeks of their life have shown very clearly that FTO contributes to the gain of body weight. This result is very similar to the data of feeding leptin-wild type mice with different *Fto* genotypes with a high caloric diet [36]. Thus, in both settings loss of FTO protects against obesity independent of the presence of leptin. However, our analysis also showed that leptin-deficient mice older than 15 weeks gain more weight than wild type independent of the *Fto* genotype. This indicates that body weight development towards obesity is delayed by about three months when FTO is absent. Based on the observation that *Fto^+/−^* mice showed a slower development of obesity on high fat diet [36] we had expected to see a similar delay for *Lep^ob/ob^;Fto^+/−^* mice. This is clearly not the case. Thus, leptin deficiency has a stronger effect than high fat diet and overrides the consequence of a 50% reduction of FTO. Nevertheless, even in mice of 30 weeks of age we can monitor a significant difference between leptin-deficient mice with and without FTO. Certainly, it would be interesting to see whether even mice one year older still show a difference. In this respect also a conditional loss of FTO would be an attractive approach to see if a deletion of FTO in leptin-deficient mice at later time points can reverse or at least slow down the further development of the obese phenotype.

### FTO and development of metabolic syndrome

We have monitored four hallmarks of the metabolic syndrome, namely glucose levels and insulin response, pancreatic islet size as well as ectopic fat accumulation. For all of these, except insulin response and insulin levels, we have found an improvement comparing leptin-deficient mice with FTO to those without FTO. Nevertheless, in contrast to the *Lep^ob/ob^* mice with FTO, the *Lep^ob/ob^* mice without FTO were able to handle high levels of glucose as showing in the GTT. Furthermore, *Lep^ob/ob^;Fto^−/−^* mice compensate insulin resistance and maintain normoglycemia without showing hypertrophy of islets of Langerhans. Recently, a study demonstrated the effect of insulin-independent glucose lowering for FGF19. Although *Lep^ob/ob^* mice were already insulin resistant, treatment with FGF19 resulted in an enhanced glucose uptake [40] indicating an insulin-independent uptake of glucose. Similarly, loss of FTO could stimulate insulin-independent glucose uptake for example of the liver, the organ which is only slightly affected by fat accumulation in the *Lep^ob/ob^;Fto^−/−^* mice. Thus, hyperglycaemia in *Lep^ob/ob^* mice is a consequence of FTO activity and leads to a stronger increase of islet size in the pancreas compared to the *Lep^ob/ob^;Fto^−/−^* mice.

The primary reason for the improvement of metabolic parameters is most likely the delay of development of obesity. Based on our analysis of food intake, *Lep^ob/ob^;Fto^−/−^* mice only consume about 20% of calories compared to *Lep^ob/ob^;Fto^+/+^* mice. Since both genotypes have a similar reduced physical activity *Lep^ob/ob^;Fto^+/+^* mice gain weight and massively develop fat depots. In addition, our analyses show that FTO deficiency in leptin deficient mice leads to a reduction of inguinal and interscapular fat tissue while the epigonadal is unaltered. Thus, FTO seems to be involved in specific fat deposition. However, comparision of epigonadal fat pads which are equal in size in *Lep^ob/ob^* mice with and without FTO revealed functional differences. Most importantly, expression of PPARγ2, the master regulator of fat-cell function, was normalised when FTO was absent. As shown in several publication (for review, see [41]), PPARγ2 has metabolic and anti-inflammatory properties leading to upregulation of adiponectin and downregulation of TNFα. Indeed, our expression analysis showed a clear tendency towards this profile as a consequence of loss of FTO. This result indicates a normalisation of at least the epigonadal adipose tissue in lipid storage and thereby a functional contribution of FTO in the development of the metabolic syndrome in addition to the kinetics of obesity development. Recently it was shown that increased energy expenditure in *Fto* negative mice might be caused by browning of epigonadal and inguinal white adipose tissue [37]. Similarly we found an upregulation of UCP-1, the marker for browning of fat tissue in *Lep^ob/ob^;Fto^−/−^* mice. Thus, although not analysed by us, increase of energy expenditure could further contribute to the improvement of the metabolic syndrome in the *Lep^ob/ob^;Fto^−/−^* mice.

Recently, several studies have addressed whether FTO in humans might be associated with the metabolic syndrome. However, the results are controversial. Whereas one study, concentrated on obese females, concluded no association [42], another using data from several studies, clearly showed an association of FTO with the metabolic syndrome [43]. Nevertheless, several studies demonstrated a clear correlation between genetic variations of the *FTO* gene and an early development of obesity, which is the main cause for the metabolic syndrome.

### Perspectives

Having shown the relevance of FTO for the development of the metabolic syndrome in an animal model, which is supported by certain GWAS in humans [43], the question arises how FTO can be a target in the context of an anti-obesity therapy. To this end, a recent publication reported about a drug used in traditional Chinese medicine called rhein, which was most efficient among several substances tested [44]. However, the assay used was exclusively based on the reduction of the demethylase activity of FTO which is the only activity described so far for FTO [45]. Similar, another study screened small molecules also used in clinical studies to block 2-OG oxygenases [46]. The limitation of both studies is that they consider the demethylase activity of FTO as relevant for its association with obesity. However, this has never been shown. Nevertheless, demethylase activity of FTO is most likely the activity being the target for an anti-obesity therapy. Thus, we are not far away to make the step from basic science to translational research.

## Supporting Information

Figure S1 **Genotype distribution at the age of 3 weeks.** 153 litters (*Lep^+/ob^;Fto^+/−^* × *Lep^+/ob^;Fto^+/−^* ) with collectively 995 offsprings are analysed and presented proportionately. Compared are the expected values with the found distribution specified for the sex. Female and male mice are underrepresented if *Fto* is completely deleted. Male *Lep^ob/ob^;Fto^−/−^* mice show an even lower survival rate than females.
(TIF)

Figure S2 **Body weight and body composition analysis of male mice.** All data are elevated from male mice. *indicate significant p-values between *Lep^ob/ob^;Fto^+/+^* and *Lep^ob/ob^;Fto^−/−^*. a) Development of body weight from 3 to 16 weeks of age. n(*Lep^+/+^;Fto^+/+^*) = 19–21, n(*Lep^ob/ob^;Fto^+/+^*) = 14–20, n(*Lep^ob/ob^;Fto^+/−^*) = 23–32, n(*Lep^ob/ob^;Fto^−/−^*) = 4–6, n(*Lep^+/+^;Fto^−/−^*) = 4–11. b) Body length at the age of 30 weeks (n = 17, 18, 28, 8, 13). c) Body weight in relation to body length at the age of 9 weeks (n = 9, 9, 9, 4, 6), 16 weeks (n = 8, 8, 9, 4, 9) an 30 weeks (n = 17, 17, 28, 8, 8). d) Lean mass and fat mass at the age of 8 weeks (n = 7, 7, 9, 2, 2). e) Fat mass in relation to lean mass at the age of 8 weeks (n = 7, 7, 9, 2, 2). All data are presented as mean. Error bars indicate the SEM.
(TIF)

Figure S3 **Metabolic data of female mice at the age of 8 weeks.** *indicate significant p-values between *Lep^ob/ob^;Fto^+/+^* and *Lep^ob/ob^; Fto^−/−^*. a) Food intake per hour. b) Food intake per hour relative to lean mass. c) Physical activity. n (a–c) = 7, 8, 4, 4, 3. Data are presented as mean. Error bars indicate the SEM.
(TIF)

Figure S4 **Liver analysis.** Organs were taken from female mice at the age of 30 weeks. *indicate significant p-values between *Lep^ob/ob^;Fto^+/+^* and *Lep^ob/ob^;Fto*. a) Liver weight (n = 13, 14, 16, 14). b) Mass of fat isolated from the liver in relation to liver weight (n = 6, 6, 6, 6). All data are presented as mean.
(TIF)
