# Supplementary figures and images for: FTO Is a Relevant Factor for the Development of the Metabolic Syndrome in Mice
Source: PLoS One. 2014 Aug 21;9(8):e105349. doi: 10.1371/journal.pone.0105349 (PMC4140775; doi:10.1371/journal.pone.0105349)

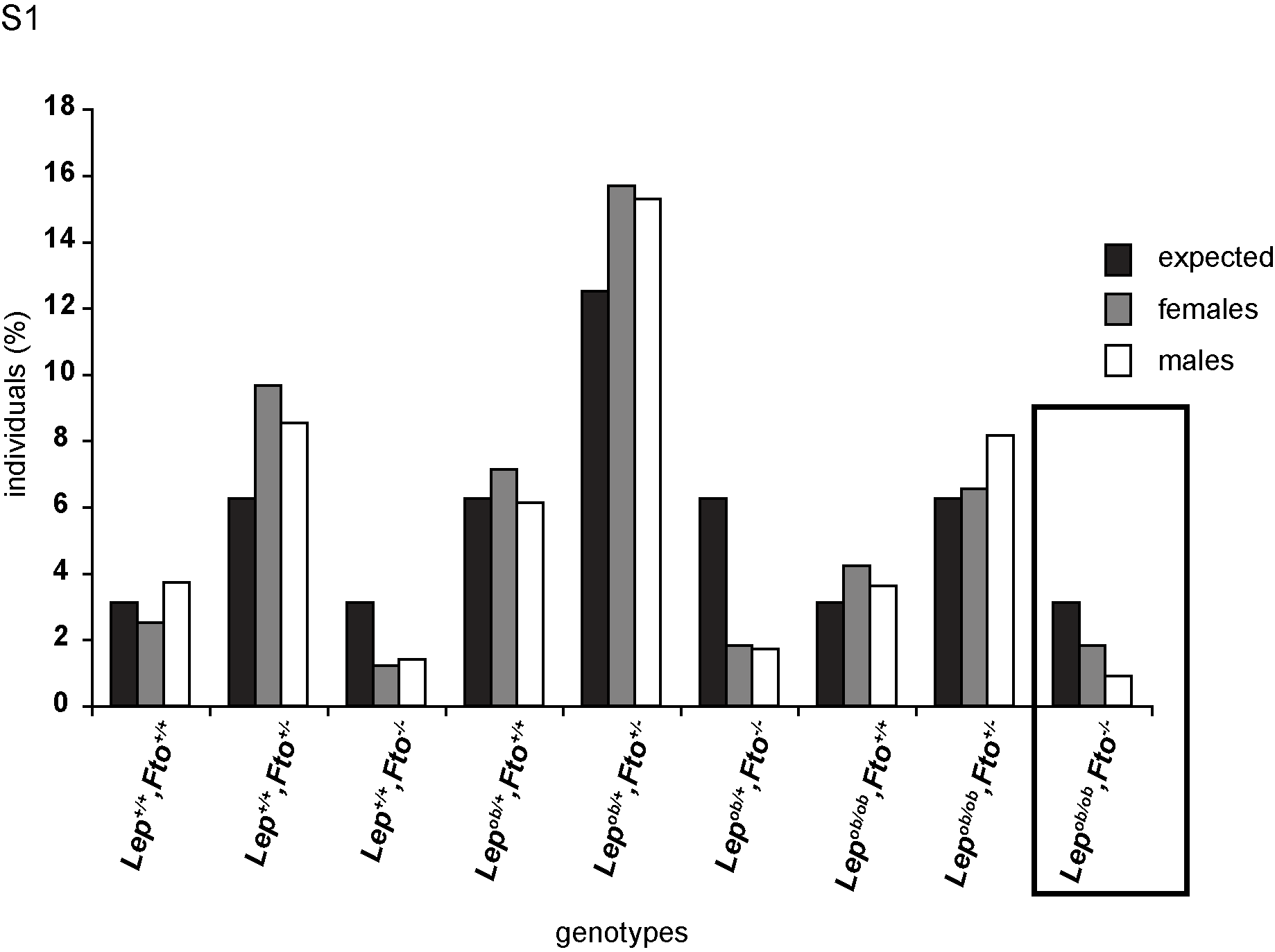

Supplement: Figure S1 — Genotype distribution at the age of 3 weeks. 153 litters (Lep+/ob;Fto+/− × Lep+/ob;Fto+/− ) with collectively 995 offsprings are analysed and presented proportionately. Compared are the expected values with the found distribution specified for the sex. Female and male mice are underrepresented if Fto is completely deleted. Male Lepob/ob;Fto−/− mice show an even lower survival rate than females. (TIF) [file pone.0105349.s001.tif]

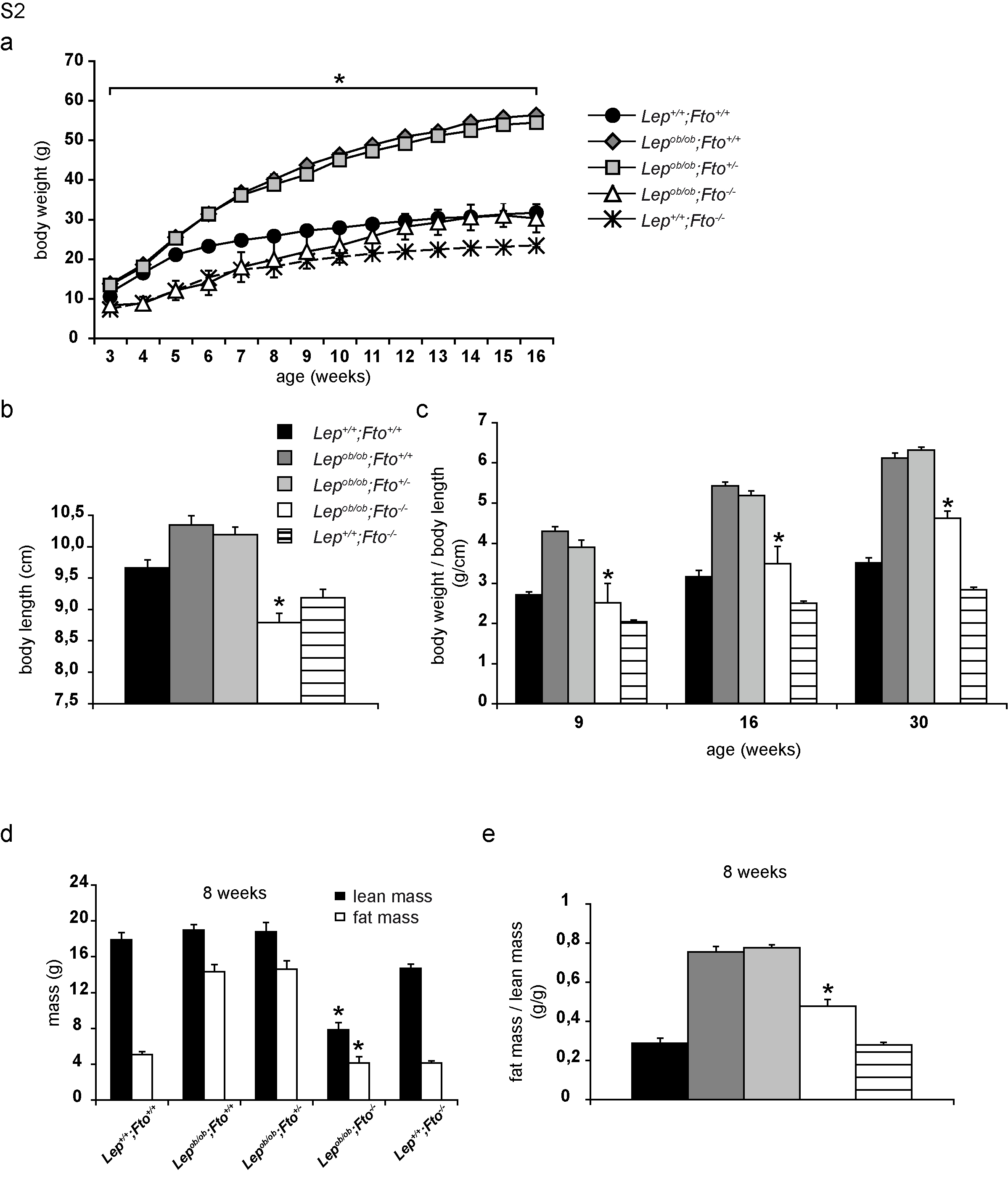

Supplement: Figure S2 — Body weight and body composition analysis of male mice. All data are elevated from male mice. *indicate significant p-values between Lepob/ob;Fto+/+ and Lepob/ob;Fto−/−. a) Development of body weight from 3 to 16 weeks of age. n(Lep+/+;Fto+/+) = 19–21, n(Lepob/ob;Fto+/+) = 14–20, n(Lepob/ob;Fto+/−) = 23–32, n(Lepob/ob;Fto−/−) = 4–6, n(Lep+/+;Fto−/−) = 4–11. b) Body length at the age of 30 weeks (n = 17, 18, 28, 8, 13). c) Body weight in relation to body length at the age of 9 weeks (n = 9, 9, 9, 4, 6), 16 weeks (n = 8, 8, 9, 4, 9) an 30 weeks (n = 17, 17, 28, 8, 8). d) Lean mass and fat mass at the age of 8 weeks (n = 7, 7, 9, 2, 2). e) Fat mass in relation to lean mass at the age of 8 weeks (n = 7, 7, 9, 2, 2). All data are presented as mean. Error bars indicate the SEM. (TIF) [file pone.0105349.s002.tif]

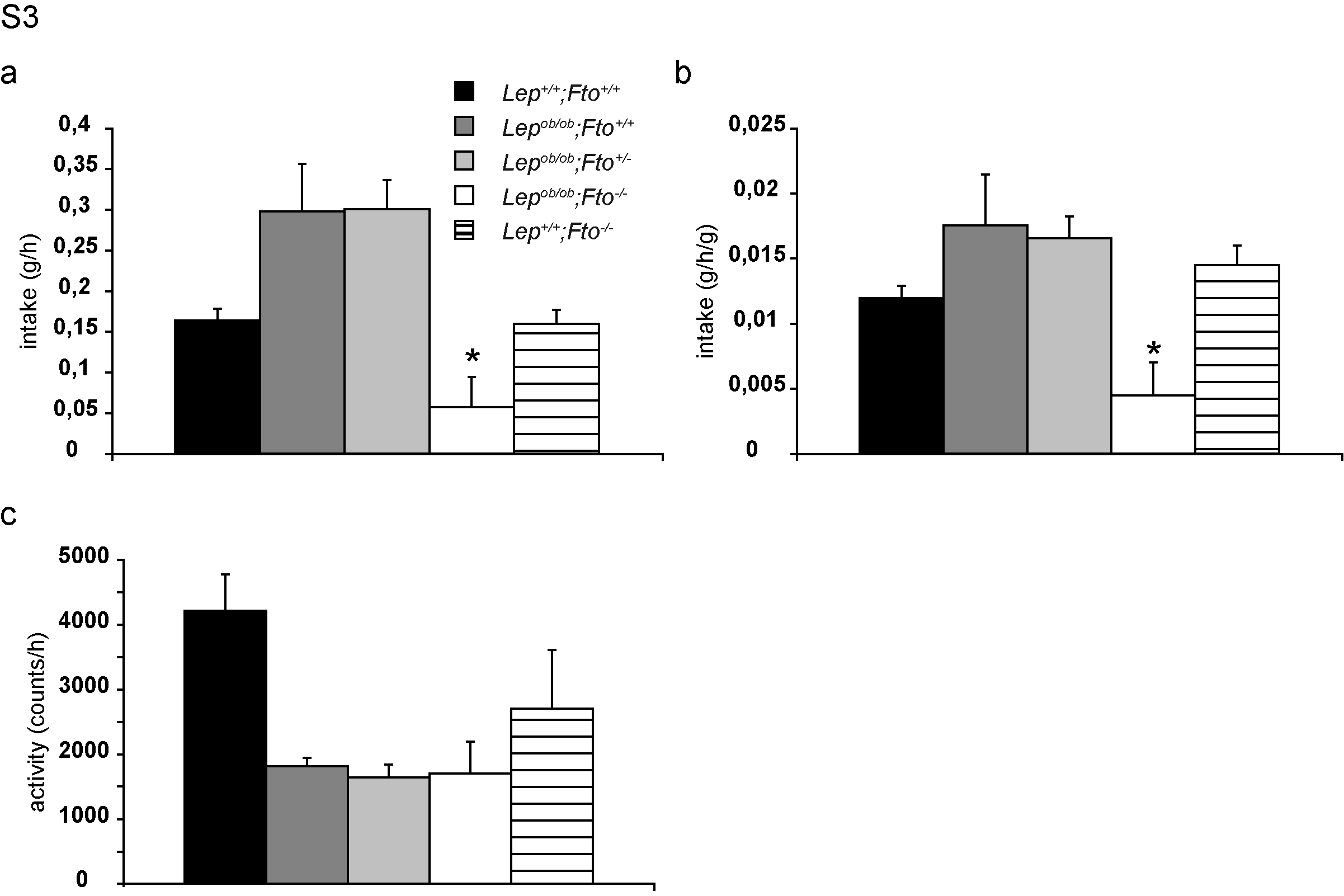

Supplement: Figure S3 — Metabolic data of female mice at the age of 8 weeks. *indicate significant p-values between Lepob/ob;Fto+/+ and Lepob/ob; Fto−/−. a) Food intake per hour. b) Food intake per hour relative to lean mass. c) Physical activity. n (a–c) = 7, 8, 4, 4, 3. Data are presented as mean. Error bars indicate the SEM. (TIF) [file pone.0105349.s003.tif]

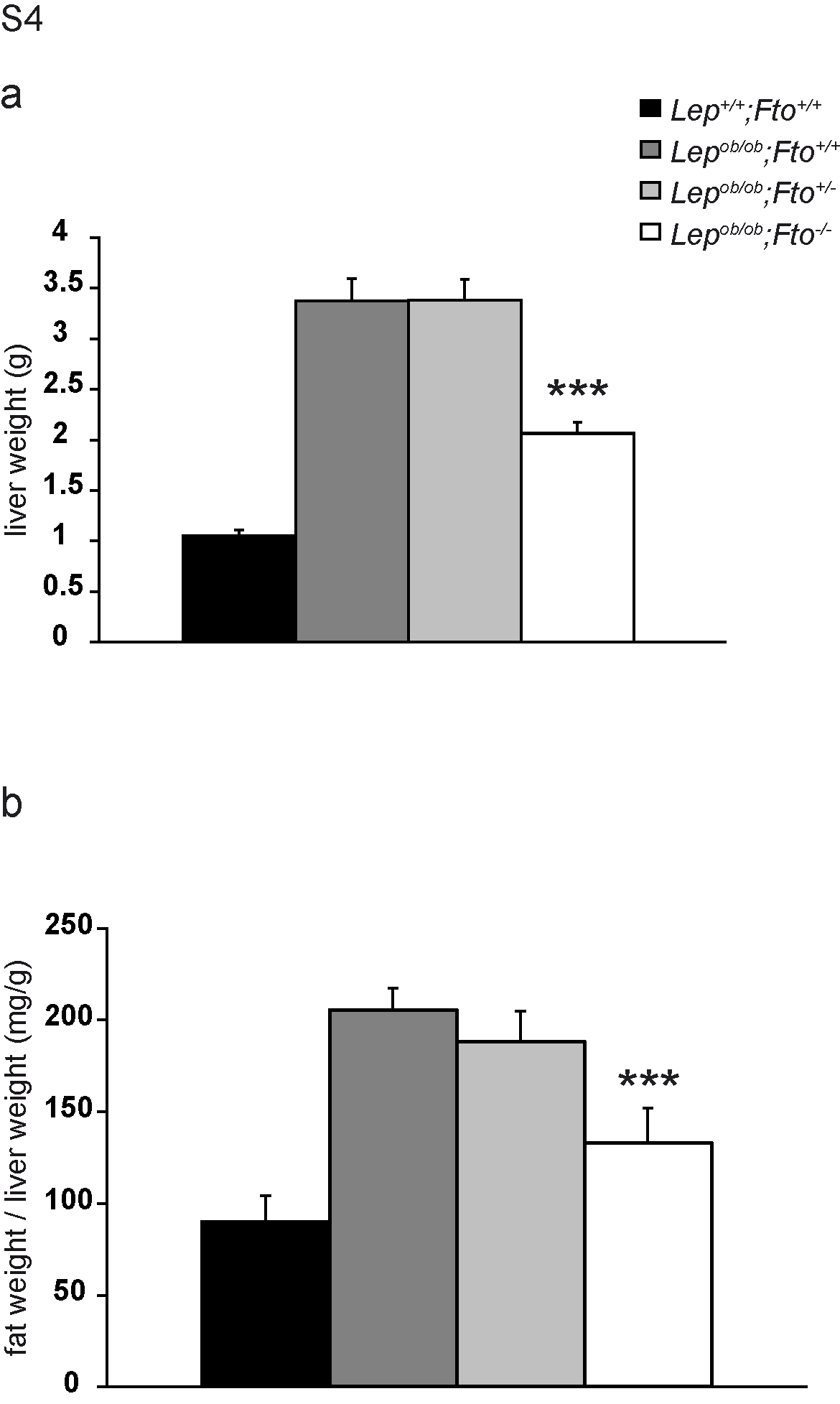

Supplement: Figure S4 — Liver analysis. Organs were taken from female mice at the age of 30 weeks. *indicate significant p-values between Lepob/ob;Fto+/+ and Lepob/ob;Fto. a) Liver weight (n = 13, 14, 16, 14). b) Mass of fat isolated from the liver in relation to liver weight (n = 6, 6, 6, 6). All data are presented as mean. (TIF) [file pone.0105349.s004.tif]
